# Botanical Gardens Are Local Hotspots for Urban Butterflies in Arid Environments

**DOI:** 10.3390/insects13100865

**Published:** 2022-09-23

**Authors:** Kathleen L. Prudic, Terese Maxine P. Cruz, Jazmyn I. B. Winzer, Jeffrey C. Oliver, Natalie A. Melkonoff, Hank Verbais, Andrew Hogan

**Affiliations:** 1 School of Natural Resources and the Environment, University of Arizona, Tucson, AZ 85721, USA; 2 Arizona Institute for Resilient Environments and Societies, University of Arizona, Tucson, AZ 85721, USA; 3 BIO5 Institute, University of Arizona, Tucson, AZ 85721, USA; 4 Department of Ecology and Evolutionary Biology, University of Arizona, Tucson, AZ 85721, USA; 5 Research Engagement, University of Arizona Libraries, Tucson, AZ 85721, USA; 6 Desert Botanical Garden, Phoenix, AZ 85008, USA; 7 Tohono Chul Botanical Gardens & Galleries, Tucson, AZ 85704, USA

**Keywords:** biodiversity, community science, conservation, pollinators, richness

## Abstract

**Simple Summary:**

Botanical gardens in urban areas can provide habitat for local wildlife. Here, we show botanical gardens, although small in total urban area, have a higher number and diversity of butterfly species than areas of similar size. Thus, botanical gardens are becoming important green refugia for pollinators and other wildlife as the climate of the southwest US continues to become warmer and drier.

**Abstract:**

Urban areas are proliferating quickly around the globe often with detrimental impacts on biodiversity. Insects, especially pollinators, have also seen record declines in recent decades, sometimes associated with land use change such as urbanization, but also associated with climate changes such as increased aridity. How these various factors play out in attracting and sustaining species richness in a complex urban matrix is poorly understood. Urban botanical gardens may serve as important refugia for insect pollinators in arid regions due to reliable water availability for both plants and insects. Here, we use community science data on butterfly observations to evaluate if botanical gardens can be hotspots of biodiversity in the arid urban landscapes of the southwest US. We found butterfly richness and diversity were proportionally overrepresented in botanical gardens compared with the urban landscape they were embedded in. We conclude that biodiversity-friendly botanical gardens in urban arid regions can make a valuable contribution to pollinator conservation, in particular, in face of the continued aridification due to climate change.

## 1. Introduction

Urbanization can negatively impact local biodiversity in a variety of taxa. Animal species richness in urban areas is generally lower than in rural areas owing to a lack of suitable habitats, habitat fragmentation, and higher levels of pesticides and pollutants [1]. However, bird species richness is often highest at intermediate levels along the urban gradient [2,3], and there are mixed reports on the relative diversity of urban insects [4]. Insect pollinator communities can respond positively to small-scale habitat features associated with nesting, host, and/or nectar resources often irrespective of larger scale land use change [5,6,7]. Urban butterflies can show both increases and decreases in species richness in urban areas relative to more wild spaces, depending on location (reviewed in: Ramírez-Restrepo and MacGregor-Fors 2016 [8,9,10]). The influential factors which promote butterfly richness and diversity estimates remain largely unknown across multiple urban locations [8,11], but are likely related to urban host and nectar availability, traffic intensity, and the baseline wildland butterfly community composition near a particular urban location.

Many insect pollinators are experiencing population declines related to climate change, resulting in wide-spread concern for their conservation and management (e.g., [12,13]). Water loss and high temperatures are physiologically challenging for small, ectothermic organisms like insect pollinators suggesting climate warming and aridification are particularly perilous for these organisms [14]. Thus, green refugia in arid lands will become increasingly important for maintaining healthy insect pollinator communities by providing microclimates and habitats within insect physiological envelopes [14]. These green refugia create local hotspots of pollinator biodiversity; here we define such hotspots as a small geographic area within a predefined larger region that exhibits high concentrations of butterfly species. Green refugia and the potential hotspots they create will be increasingly found in urban environments as more water is allocated to the needs of human populations than agriculture and wildlands (e.g., [15,16]). Although cities are not typically built as habitat for local plants and animals, they do contain important wildlife habitats, such as parks, nature preserves, golf courses, cemeteries, and yards [8,9] and there are many opportunities for additional, functional habitat creation within urban areas, including for migratory and threatened species [17]. Insect pollinators have limited habitat ranges, relatively short life cycles, and require minimal space for foraging and nesting as compared to many other species of conservation concern [18]. Thus, these relatively small spatial and temporal habitat requirements make the creation of urban habitat not only manageable but also critical in the face of pollinator decline. Additionally, efforts to incorporate natural habitats into urban planning are increasingly more common and driven by conservation, human health, and climate change mitigation awareness and needs [9,19].

Botanical gardens provide multiple benefits to their communities (e.g., [20]). Their mission is to promote the awareness, scientific study, and conservation of plant species diversity. They typically cultivate a combination of exotic and native plant species and host a variety of activities including conservation, propagation, horticulture, seed science, taxonomy, systematics, genetics, biotechnology, restoration ecology, and formal education [21,22]. They house both living collections and herbaria that contain valuable data on plant distribution, diversity, and genetic material that can serve as the baseline for numerous studies and species assessments [21]. Botanical gardens also provide other intrinsic value such as nature driven mental health support, human connections, art displays and exhibits, and informal science education opportunities for the visitors they engage with [22,23]. Less well understood is the role botanical gardens play in supporting local wildlife including insect pollinators [20,24]. Consistent availability of plants and water resources create potentially important green refugia and hotspots for local insect pollinators within a complex urban matrix [20,24].

Here, we compare butterfly species richness and diversity estimates in the botanical gardens of five urban areas in the southwest area of the United States. Based on observational data derived primarily from the eButterfly [25] and iNaturalist [26] community science projects, we compare butterfly species richness and diversity of gardens to butterfly species richness and diversity of the larger metropolitan area in which the gardens are located. We hypothesize botanical gardens will have a high proportion of total urban butterfly diversity within a relatively small area. We use permutation analyses to create appropriate comparisons between these gardens and corresponding larger urban areas. We discuss the implications of the results on butterfly species, the botanical gardens, and how botanical gardens can provide green refugia to local wildlife in addition to their other benefits.

## 2. Methods

### 2.1. Botanical Gardens and Cities

To investigate the potential of urban botanical gardens serving as butterfly hotspots, we focused on five large, urban cities of the southwestern United States of America: Tucson and Phoenix, Arizona; Palm Desert, California; Albuquerque, New Mexico; and El Paso, Texas. All the cities average less than 11 inches of precipitation annually [27] and, with the exception of Palm Desert, CA, each has a population over 500,000 residents. Each city includes at least one botanical garden focused on native and arid-adapted plants (Table 1). We selected botanical gardens that had over 40 community science observations of butterflies (see below). This criterion excluded one additional city in the arid southwest, Las Vegas, Nevada, and one botanical garden from El Paso, TX, due to insufficient community science records.

### 2.2. Community Science Data

We used records of butterflies collected through two community science efforts: the eButterfly platform, which exclusively focuses on butterfly observations, and iNaturalist, which includes observational records of all taxa. We used the R programming language [28] and the R package rgbif [29] to download data from the Global Biodiversity Informatics Facility (https://gbif.org (accessed on 21 June 2022)). Data were vetted by both eButterfly and iNaturalist in a variety of ways [25,26] respectively before submission to GBIF and taxonomic differences were reconciled by GBIF. iNaturalist data for this study were restricted to those categorized as Research Grade. We downloaded data for each garden, based on geographic coordinates that defined a rectangle that included the botanical garden. We downloaded data for each city based on city boundaries available through the OpenStreetMap project with the R packages osmdata [30] and sf [31] and limited our data set to observations made between 1 January 2000 and 21 June 2022.

### 2.3. Statistical Analyses

For each garden, we sought to determine if the richness and diversity of butterfly species observed in the garden were higher than the same measures in the corresponding city. Species richness is defined as the total number of species observed and we used Shannon’s index as our measure of species diversity [32]. Before calculations, we removed duplicated samples, based on geographical coordinates, date, and species identification. We first calculated the species richness and diversity for each garden, using the number of observations of a species as the proxy for abundance in diversity calculations. To compare garden richness and diversity to that of the corresponding cities, we performed repeated sampling to generate distributions of city richness and diversity, similar to the approach described in [33]. For each permutation, we selected an area of the city defined by a rectangle of the same dimensions as that for the garden in question. As significant portions of the cities lack any observational data, we did not select areas completely at random. Rather, we used all unique pairs of geographical coordinates of observations within the city to serve as centroids for a sampling rectangle. We randomly sampled rectangles defined by those coordinates, with replacement, and all observations that fell within that rectangle were used to calculate one sample of richness and diversity for the city. For each garden, we performed 1000 sampling replicates to generate a distribution of richness and diversity for each city. To provide a proper comparison between gardens and cities, we excluded all observations from within the garden when calculating sample richness and diversity for the city. We then compared the scalar garden richness and diversity values to the distributions of the corresponding city. When comparing garden measures of richness and diversity to that of the corresponding city, we report the values for the garden as a percentile of the values from the distribution of 1000 sampling replicates. The quantitative criteria of a hotspot is an ongoing discussion within and between taxa [34]. Here, we assessed an urban butterfly hotspot *a priori* as a value above the 75th percentile of permutation replicates, and we reported the values for each site for future reference and comparisons. All analyses were performed with the R programming language [28], with the aid of the dplyr [35] and tidyr [36] packages. Additionally, we used the ggplot2 [37], ggpubr [38], and stringr [39] R packages for data visualization. All code for data collection, analysis, and visualization is available at (https://doi.org/10.5281/zenodo.7065737 accessed on 21 June 2022).

## 3. Results

### 3.1. Community Science Data

**Figure 1:**
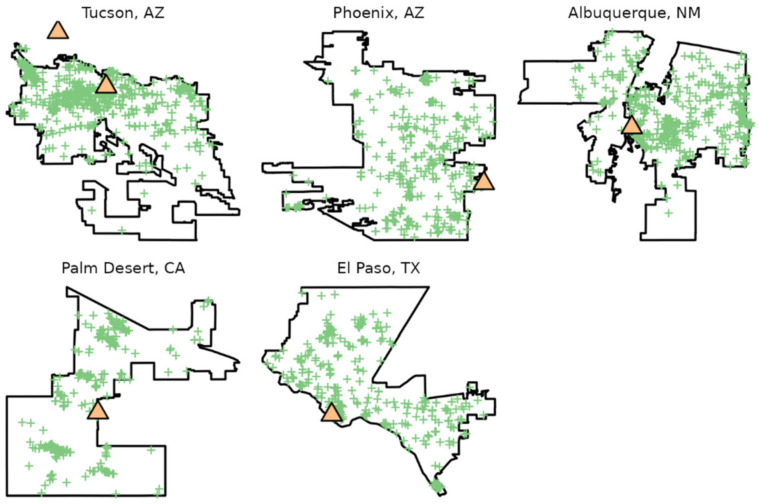
Locations of boundaries, botanical gardens (orange triangles), and community science observations (green crosses) used in the current study. City boundaries based on data from OpenStreetMap (2017).

The resulting data set for the six gardens and five cities included 10,014 total observations after de-duplication of records (Figure 1 and Table 2), including 121 species (Tables S1 and S2). e-Butterfly had 4471 observations while iNaturalist had 7718 observations. The total from the two community science data was greater than the number of records after de-duplication because some records are present in both data sets.

### 3.2. Species Richness and Diversity

**Figure 2:**
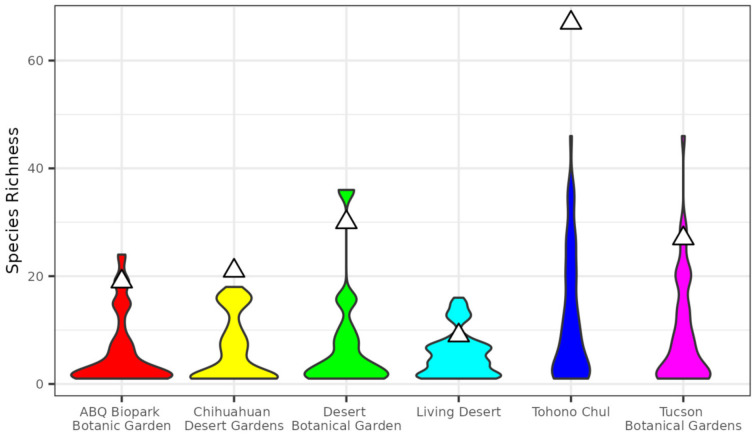
Comparison of species richness in botanical gardens to samples of identical geographic size within city boundaries. Violins show distribution of richness based on 1000 replicates and triangles show observed species richness for corresponding gardens.

Species richness in the botanical gardens was generally higher than observational samples from the corresponding city (Figure 2). Species richness in gardens was in the 86th percentile or above of permutation results, indicating proportional overrepresentation of species in botanical gardens meeting our hotspot criteria of above the 75th percentile (Table 3). Two gardens, Tohono Chul of Tucson, AZ, and the Chihuahuan Desert Garden of El Paso, TX, had species richness higher than all permutation replicates of the corresponding city.

**Figure 3:**
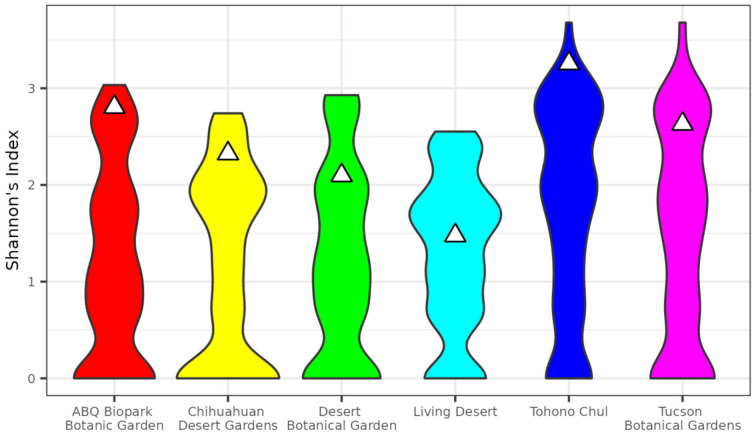
Comparison of species diversity in botanical gardens to samples of identical geographic size within city boundaries. Violins show distribution of Shannon’s index based on 1000 replicates and triangles show observed values of Shannon’s index for corresponding gardens.

Species diversity, as measured by Shannon’s Index, were also proportionally higher in gardens than in the corresponding city (Figure 3). With the exception of the Living Desert in Palm Desert, CA, botanical gardens’ diversity were in the 76th percentile or above of permutation results (Table 3). Two botanical gardens, Tohono Chul of Tucson, AZ and the ABQ Biopark Botanic Garden of Albuquerque, NM, were above the 95 percentile of permutation results.

## 4. Discussion

Community scientists reported over 10,500 butterfly observations in the five cities from 2000–2022 to eButterfly and iNaturalist that were pushed to GBIF (Table 1). The observed botanical gardens had disproportionately higher butterfly richness and diversity for their size. Species richness of gardens ranged in the 86–100 percentiles across permutation replicates (Figure 2, Table 2). We expected botanical gardens to be around the 50th percentile if they were capturing the same richness as the rest of the city. Instead, the species richness scores were high enough at all botanical gardens to reach our hotspot criteria, or >75th percentile. Two gardens, Tohono Chul of Tucson, AZ, and the Chihuahuan Desert Garden of El Paso, TX, had species richness higher than all permutation replicates of the corresponding city (Table 2). Species diversity, as measured by Shannon’s Index, were also disproportionately higher in botanical gardens than in the corresponding city. Here, too, we expected botanical gardens to be around the 50th percentile if they were capturing the same diversity as the rest of the city. Botanical garden species diversity percentile ranged from 76–98 across permutation replicates in 5 of the 6 botanical gardens meeting the hotspot criteria. One botanical garden, the Living Desert Botanical Garden of Palm Desert, CA was in the 53rd percentile of permutation replicates and did not meet hotspot criteria (Figure 3, Table 3). Two of the six botanical gardens, Tohono Chul and The Living Desert, warrant special discussion given the results.

The Tohono Chul Botanical Garden in Tucson, AZ, was the only garden with more observations than in the corresponding city (Table 3). This is largely driven by the butterfly monitoring effort this garden performs with its volunteers and community members. The docents at Tohono Chul, two of which are co-authors on this paper, run a weekly 2-h butterfly walk where all observed species are reported to eButterfly.org. These efforts have been ongoing for the past 8 years. Many of the volunteers have also participated in the Master Naturalist program and are trained on other community science platforms such as eBird [40] and Nature’s Notebook [41] so they are frequent contributors to biodiversity community science projects. As a garden activity, these butterfly walks and the other community science programming have been an instrumental point of contact for connecting current volunteers, training, and attracting new volunteers, and educating the public on pollinators and their conservation. These walks are also providing biodiversity data and data products to city planners and urban conservation efforts such as Trees for Tucson (https://tucsoncleanandbeautiful.org/trees-for-tucson (accessed on 21 June 2022)) and the Santa Cruz River Heritage Project (https://www.tucsonaz.gov/water/SCRHP (accessed on 21 June 2022)).

The Living Desert Botanical Garden in Palm Springs, CA, had the lowest percentile of species diversity of any botanical garden in the study (52 percentile of permutation results, Table 3). In this case, the government city boundaries likely affected the results: the incorporated area of Palm Springs includes large greenspaces starting at the base of the San Jacinto Mountains and extends across a substantial elevational gradient (~950 m in elevation gain over undeveloped land). This led to a higher estimate of species richness and diversity at the city level due to the inclusion of a significant number of non-urban locations. Other factors likely influence our estimates of butterfly richness and diversity across all urban areas studied here including traffic volume, water availability in and outside the gardens, pesticide usage, and native plant richness and abundance warranting future study. Future work should include additional attention on how these factors influence spatial diversity patterns in the urban landscape.

Here, we found evidence that botanical gardens support more butterflies than other areas of similar size in urban areas of the arid southwest and meet our criteria for urban butterfly hotspots. Continued monitoring efforts in botanical gardens is warranted and needed by scientists, urban planners, and conservation managers as the world changes. These community science efforts should be expanded to other pollinator groups including bats, bees, and hummingbirds. Additional efforts to record plant affiliations through iNaturalist would also be helpful and could be accomplished by submitting a photograph twice: once for the animal, once for the plant. Given current climate projections, we anticipate botanical gardens and other green refugia will be critical habitat for urban wildlife as water becomes less available and redistributed across the landscape. Encouraging and coordinating botanical volunteers to monitor the pollinators in other urban greenspaces such as parks, schools, cemeteries, and community gardens would be helpful to understand and predict urban ecosystem function, climate mitigation, and restoration efforts. Botanical gardens can help promote coexistence and guide future interactions between humans and wildlife in cities, helping to guide urban conservation and management efforts while continuing to educate the public on the importance and needs of urban wildlife.

## 5. Conclusions

Botanical gardens are important urban habitats for pollinators and other local wildlife [42]. We estimate botanical gardens are hotspots for butterflies in arid cities. Botanical gardens, despite covering a very small percentage of city area (0.002–0.22%), had disproportionately high butterfly species richness and diversity compared with the much larger surrounding city area (>75th percentile of permutation replicates). Continued monitoring of butterflies, bees, bats, and hummingbirds in these spaces will be essential for understanding the impacts of climate change and animal biodiversity in urban spaces while providing a way for community members to connect with conservation efforts. Improved understanding of animal biodiversity patterns in botanical gardens can help communities better manage green infrastructure investments, which are important for improving ecological health, community livability, and environmental equity within urban areas. Botanical gardens and the plants they support are hotspot habitats for local urban butterfly species, now and in the quickly changing future.

## Figures and Tables

**Table 1:** Location and areas of botanical gardens included in this study. Areas are measured in square kilometers. The column “% City Area” is the size of the garden relative to the size of the corresponding city.

City, State	Botanical Garden	City Area	Garden Area	% City Area
Phoenix, AZ	Desert Botanical Garden	1374.354	0.367	0.0267
Tucson, AZ	Tohono Chul	590.61	0.067	0.0113
	Tucson Botanical Garden	590.61	0.021	0.0036
Palm Desert, CA	Living Desert	154.304	0.347	0.2246
Albuquerque, NM	ABQ BioPark Botanic Garden	525.763	0.271	0.0516
El Paso, TX	Chihuahuan Desert Gardens	710.393	0.012	0.0016

**Table 2:** Total number of unique observations for each city and botanical garden.

City, State	Botanical Garden	Garden Observations	City Observations
Phoenix, AZ	Desert Botanical Garden	283	1424
Tucson, AZ	Tohono Chul	4227	2142
	Tucson Botanical Gardens	132	
Palm Desert, CA	Living Desert	51	420
Albuquerque, NM	ABQ BioPark Botanic Garden	40	801
El Paso, TX	Chihuahuan Desert Gardens	106	1000

**Table 3:** Species richness and diversity (Shannon’s Index) for each botanical garden. Percentiles are observed richness and diversity values relative to 1000 permutation sampling replicates in the corresponding city.

Garden	Richness	City Richness	Richness Percentile	Diversity	City Diversity	Diversity Percentile
Desert Botanical Garden	30	51	91.2	2.1	2.95	84.5
Tohono Chul	67	79	100	3.26	3.41	98
Tucson Botanical Gardens	27	79	92	2.63	3.41	76.3
Living Desert	9	28	85.8	1.48	2.72	52.7
ABQ Biopark Botanic Garden	19	61	95.4	2.8	3.26	95.4
Chihuahuan Desert Gardens	21	54	100	2.32	3.12	85.2

## Data Availability

Data used in this work, along with all source codes for data processing and analysis, are available on GitHub at https://github.com/Big-Biodiversity-Collaborative/BotanicGardenHotspot (accessed on 21 June 2022). Additionally, data and source code are also archived at Zenodo (https://doi.org/10.5281/zenodo.7065737 (accessed on 21 June 2022)).

## Supplementary Materials

The following supporting information can be downloaded at: https://www.mdpi.com/article/10.3390/insects13100865/s1, Table S1: Butterfly species observed in six botanical gardens; Table S2: Butterfly species observed in five cities.
