# Supplementary material for: Botanical Gardens Are Local Hotspots for Urban Butterflies in Arid Environments
Source: Insects. 2022 Sep 23;13(10):865. doi: 10.3390/insects13100865 (PMC9604306; doi:10.3390/insects13100865)
Supplement: Supplementary file 1 [file insects-13-00865-s001.zip › insects-1892938-supplementary.pdf]

Supplemental Tables

Table S1. Butterfly species observed in six botanical gardens in southwestern USA from January 1, 2000 and June 21, 2022. Data was aggregated from iNaturalist and eButterfly via the Global Biodiversity Informatics Facility (GBIF) on June 22, 2022. Names are from GBIF backbone taxonomy (https://doi.org/10.15468/39omei). Duplicate observations were removed before data summary and analysis. Data and R code for data cleaning and analyses are archived in a publicly accessible repository, Zenodo https://doi.org/10.5281/zenodo.7065737. This information can also be found on GitHub, https://github.com/Big-Biodiversity-Collaborative/BotanicGardenHotspot.

Family	Species	Desert Botanical Garden	Tohono Chul	Tucson Botanical Gardens	Living Desert	ABQ BioPark Botanic Garden	Chihuahuan Desert Gardens
Hesperiidae	*Chiomara georgina*		4				
Hesperiidae	*Cogia hippalus*		3				
Hesperiidae	*Copaeodes aurantiaca*		5	1			
Hesperiidae	*Erynnis funeralis*	1	37				2
Hesperiidae	*Erynnis tristis*		11				
Hesperiidae	*Heliopyrgus domicella*		1				
Hesperiidae	*Hylephila phyleus*	2	52	5	1	1	2
Hesperiidae	*Lerodea eufala*		18				1
Hesperiidae	*Pholisora catullus*		4				
Hesperiidae	*Pyrgus communis*		14				
Hesperiidae	*Systasea zampa*	1	23	1			
Hesperiidae	*Urbanus dorantes*		2				
Lycaenidae	*Apodemia palmerii*		19				

Lycaenidae	*Atlides halesus*		25				
Lycaenidae	*Brephidium exilis*	2	5				7
Lycaenidae	*Calephelis arizonensis*		2				
Lycaenidae	*Calephelis nemesis*	1	107	2			
Lycaenidae	*Callicista columella*		1				
Lycaenidae	*Celastrina ladon*		30				
Lycaenidae	*Chlorostrymon simaethis*		3				
Lycaenidae	*Echinargus isola*	3	31				1
Lycaenidae	*Hemiargus ceraunus*	13	60	4			3
Lycaenidae	*Leptotes marina*	7	300	8	4	1	7
Lycaenidae	*Ministrymon leda*	2	16	1			
Lycaenidae	*Mitoura siva*			1			
Lycaenidae	*Strymon melinus*	10	74	5	1	2	2
Nymphalidae	*Agraulis vanillae*		295				
Nymphalidae	*Anthanassa texana*	4	263	19		1	1
Nymphalidae	*Asterocampa celtis*		2	1			
Nymphalidae	*Asterocampa leilia*	6	175	5			
Nymphalidae	*Chlosyne lacinia*		12				
Nymphalidae	*Cynthia annabella*	2	2	1	1		1
Nymphalidae	*Danaus gilippus*	152	376	27	24	1	42
Nymphalidae	*Danaus plexippus*	23	261	20	15	3	1
Nymphalidae	*Dymasia dymas*		40	1			

Nymphalidae	*Eresia aveyrana*					1	
Nymphalidae	*Euptoieta claudia*		6	1		2	
Nymphalidae	*Heliconius charithonia*	1	1				
Nymphalidae	*Junonia coenia*		5				
Nymphalidae	*Junonia genoveva*		1				
Nymphalidae	*Libytheana carinenta*	9	165	2			5
Nymphalidae	*Limenitis arthemis*	1					
Nymphalidae	*Limenitis bredowii*		1				
Nymphalidae	*Nymphalis antiopa*		1			3	
Nymphalidae	*Oeneis polixenes*		1				
Nymphalidae	*Phyciodes tharos*					2	
Nymphalidae	*Polygonia interrogationis*					3	
Nymphalidae	*Texola elada*		20				
Nymphalidae	*Thessalia leanira*		1				
Nymphalidae	*Vanessa atalanta*	2	5			1	
Nymphalidae	*Vanessa cardui*	15	83	8	2	3	
Nymphalidae	*Vanessa virginiensis*		4				
Papilionidae	*Battus philenor*	11	310	1			5
Papilionidae	*Papilio astyalus*		1				
Papilionidae	*Papilio multicaudata*		1			4	8
Papilionidae	*Papilio polibetes*		2	1		4	1
Papilionidae	*Papilio rumiko*	2	160	11			3

Pieridae	*Abaeis nicippe*	11	238			1	7
Pieridae	*Anthocharis cethura*		1				
Pieridae	*Anthocharis sara*		1				
Pieridae	*Ascia monuste*	1	1				
Pieridae	*Colias eurytheme*	1	18			1	
Pieridae	*Eurema boisduvaliana*		6				
Pieridae	*Eurema mexicana*		168				
Pieridae	*Kricogonia lyside*	2	8				
Pieridae	*Nathalis iole*	6	206		2		1
Pieridae	*Phoebis agarithe*		50		1		
Pieridae	*Phoebis sennae*	1	237	1			3
Pieridae	*Pieris rapae*		1	3		2	
Pieridae	*Pontia protodice*	3	74	1		4	3
Pieridae	*Pyrisitia nise*			1			
Pieridae	*Pyrisitia proterpia*		22				
Pieridae	*Zerene cesonia*	1	167	1			

Table S2. Butterfly species observed in five cities in southwestern USA from January 1, 2000 and June 21, 2022. Data was aggregated from iNaturalist and eButterfly via the Global Biodiversity Informatics Facility (GBIF) on June 22, 2022. Names are from GBIF backbone taxonomy (https://doi.org/10.15468/39omei). Duplicate observations were removed before data summary and analysis. Data and R code for data cleaning and analyses are archived in a publicly accessible repository, Zenodo https://doi.org/10.5281/zenodo.7065737. This information can also be found on GitHub, https://github.com/Big-Biodiversity-Collaborative/BotanicGardenHotspot.

Family	Species	Phoenix, AZ	Tucson, AZ	Palm Desert, CA	Albuquerque, NM	El Paso, TX
Hesperiidae	*Amblyscirtes nysa*		1			1
Hesperiidae	*Ancyloxypha arene*		1		3	4
Hesperiidae	*Atalopedes campestris*		1			2
Hesperiidae	*Atrytonopsis vierecki*					1
Hesperiidae	*Chiomara georgina*		1			
Hesperiidae	*Copaeodes aurantiaca*		17	1	1	
Hesperiidae	*Epargyreus clarus*				1	
Hesperiidae	*Erynnis brizo*				2	2
Hesperiidae	*Erynnis funeralis*	10	12	11	5	8
Hesperiidae	*Erynnis juvenalis*		1			
Hesperiidae	*Erynnis telemachus*				1	
Hesperiidae	*Erynnis tristis*	2	2			2
Hesperiidae	*Heliopetes ericetorum*	5		1		
Hesperiidae	*Heliopyrgus domicella*	2				
Hesperiidae	*Hesperia pahaska*				2	
Hesperiidae	*Hylephila phyleus*	91	106	25	34	23
Hesperiidae	*Lerodea arabus*		4			
Hesperiidae	*Lerodea eufala*	7	28			12
Hesperiidae	*Pholisora catullus*	1	6			2

Hesperiidae	*Poanes taxiles*				1	
Hesperiidae	*Pyrgus communis*				3	
Hesperiidae	*Pyrgus oileus*		1			
Hesperiidae	*Pyrgus scriptura*		8			
Hesperiidae	*Staphylus ceos*		5			
Hesperiidae	*Systasea zampa*	8	11			1
Hesperiidae	*Zestusa dorus*				1	
Lycaenidae	*Apodemia mormo*			5	1	5
Lycaenidae	*Apodemia palmerii*		7			
Lycaenidae	*Atlides halesus*	10	14		4	3
Lycaenidae	*Brephidium exilis*	61	103	19	53	84
Lycaenidae	*Calephelis nemesis*	3	31			
Lycaenidae	*Calephelis wrighti*			9		
Lycaenidae	*Callicista columella*	2	1			
Lycaenidae	*Callophrys spinetorum*				1	
Lycaenidae	*Celastrina ladon*	2	3		2	
Lycaenidae	*Echinargus isola*	20	16	3	18	5
Lycaenidae	*Euphilotes bernardino*			1		
Lycaenidae	*Hemiargus ceraunus*	41	41	35	2	11
Lycaenidae	*Icaricia lupini*		4		4	
Lycaenidae	*Leptotes marina*	43	91	30	33	59
Lycaenidae	*Ministrymon leda*	4	36			1

Lycaenidae	*Mitoura gryneus*	1	1		1	
Lycaenidae	*Mitoura siva*		6			
Lycaenidae	*Sandia mcfarlandi*				39	
Lycaenidae	*Strymon melinus*	57	82	23	22	29
Nymphalidae	*Agraulis vanillae*		6			
Nymphalidae	*Anaea aidea*					3
Nymphalidae	*Anaea andria*				1	
Nymphalidae	*Anthanassa texana*	6	61		2	17
Nymphalidae	*Asterocampa celtis*		23			
Nymphalidae	*Asterocampa leilia*	28	60			
Nymphalidae	*Cercyonis pegala*				1	
Nymphalidae	*Chlosyne californica*	12	1	1		
Nymphalidae	*Chlosyne lacinia*	2	5		5	10
Nymphalidae	*Cyllopsis pertepida*				1	
Nymphalidae	*Cynthia annabella*	27	32		5	11
Nymphalidae	*Danaus gilippus*	353	289	67	13	182
Nymphalidae	*Danaus plexippus*	81	120	32	28	30
Nymphalidae	*Dryas iulia*				1	
Nymphalidae	*Dymasia dymas*	2	14	9		
Nymphalidae	*Eresia aveyrana*				3	
Nymphalidae	*Euptoieta claudia*	18	17		37	19
Nymphalidae	*Gyrocheilus patrobas*		1			

Nymphalidae	*Heliconius charithonia*	1				
Nymphalidae	*Junonia coenia*				1	
Nymphalidae	*Junonia genoveva*				1	
Nymphalidae	*Junonia nigrosuffusa*		4		1	2
Nymphalidae	*Libytheana carinenta*	149	111	6	19	17
Nymphalidae	*Limenitis archippus*	2	1		2	
Nymphalidae	*Limenitis arthemis*	1	2			
Nymphalidae	*Limenitis bredowii*		2		8	1
Nymphalidae	*Limenitis weidemeyerii*				1	
Nymphalidae	*Marpesia petreus*		1			
Nymphalidae	*Microtia elva*		2			
Nymphalidae	*Nymphalis antiopa*	3	11		36	3
Nymphalidae	*Occidryas chalcedona*			2		
Nymphalidae	*Oeneis polixenes*		4			1
Nymphalidae	*Phyciodes picta*					2
Nymphalidae	*Phyciodes tharos*				14	2
Nymphalidae	*Poladryas minuta*		1			
Nymphalidae	*Polygonia interrogationis*		1		5	5
Nymphalidae	*Siproeta stelenes*					1
Nymphalidae	*Texola perse*		4			
Nymphalidae	*Thessalia leanira*				2	
Nymphalidae	*Thessalia theona*					2

Nymphalidae	*Vanessa atalanta*	29	11		7	12
Nymphalidae	*Vanessa cardui*	157	197	87	88	54
Nymphalidae	*Vanessa virginiensis*	4	6		2	3
Papilionidae	*Battus philenor*	26	81		1	17
Papilionidae	*Papilio multicaudata*		4		117	105
Papilionidae	*Papilio polibetes*	4	15		22	29
Papilionidae	*Papilio rumiko*	32	135	4		35
Papilionidae	*Papilio rutulus*				4	
Papilionidae	*Papilio zelicaon*		1			
Pieridae	*Abaeis nicippe*	187	96	1	12	56
Pieridae	*Anthocharis cethura*	1	2	1		1
Pieridae	*Anthocharis sara*	1		4		
Pieridae	*Anthocharis thoosa*		1		2	2
Pieridae	*Ascia monuste*	1	10			2
Pieridae	*Colias eurytheme*	12	13		14	8
Pieridae	*Colias philodice*		1		9	
Pieridae	*Euchloe lotta*		1			
Pieridae	*Eurema mexicana*		1			
Pieridae	*Kricogonia lyside*	2	5			
Pieridae	*Nathalis iole*	104	52	11	34	30
Pieridae	*Phoebis agarithe*	6	6	11		
Pieridae	*Phoebis sennae*	27	53	3		16

Pieridae	*Pieris rapae*		9		40	11
Pieridae	*Pontia beckerii*			10		
Pieridae	*Pontia protodice*	57	53	19	47	71
Pieridae	*Pontia sisymbrii*				3	
Pieridae	*Pyrisitia nise*		4			
Pieridae	*Pyrisitia proterpia*	3	15			1
Pieridae	*Zerene cesonia*	4	32		1	7
